# Effectiveness of Switching CGRP Monoclonal Antibodies in Non-Responder Patients in the UAE: A Retrospective Study

**DOI:** 10.3390/neurolint16010019

**Published:** 2024-02-18

**Authors:** Reem Suliman, Vanessa Santos, Ibrahim Al Qaisi, Batool Aldaher, Ahmed Al Fardan, Hajir Al Barrawy, Yazan Bader, Jonna Lyn Supena, Kathrina Alejandro, Taoufik Alsaadi

**Affiliations:** American Center for Psychiatry and Neurology, Abu Dhabi P.O. Box 108699, United Arab Emiratesbatool.c.2014@gmail.com (B.A.);

**Keywords:** CGRP monoclonal antibodies, effectiveness, migraine, treatment, switching

## Abstract

Calcitonin gene-related peptide monoclonal antibodies (CGRP mAbs) have shown promising effectiveness in migraine management compared to other preventative treatment options. Many questions remain regarding switching between antibody classes as a treatment option in patients with migraine headaches. This preliminary retrospective real-world study explored the treatment response of patients who switched between CGRP mAb classes due to lack of efficacy or poor tolerability. A total of 53 patients with migraine headache switched between three of the CGRP mAbs types due to lack of efficacy of the original prescribed CGRP mAbs, specifically eptinezumab, erenumab, and galcanezumab. Fremanezumab was not included due to unavailability in the UAE. Galcanezumab and eptinezumab target the CGRP ligand (CGRP/L), while erenumab targets CGRP receptors (CGRP/R). The analysis of efficacy demonstrated that some improvements were seen in both class switch cohorts (CGRP/R to CGRP/L and CGRP/L to CGRP/R). The safety of switching between CGRP classes was well observed, as any adverse events presented before the class switch did not lead to the discontinuation of treatment following the later switch. The findings of this study suggest that switching between different classes of CGRP mAbs is a potentially safe and clinically viable practice that may have some applications for those experiencing side effects on their current CGRP mAb or those witnessing suboptimal response.

## 1. Introduction

Migraine is a neurological disorder experienced by an estimated global point prevalence range of 14,107 cases per 100,000 [1]. According to the 2019 Global Burden of Disease Study, migraine is the second most common non-fatal disease in terms of “years lived with disability” [2]. Those suffering from migraine experience a significant impact on their ability to maintain their productivity and relationships, mandating continuous efforts to better understand its pathophysiology and optimal treatment options [3]. Although the precise mechanism of migraine remains unknown, recent findings suggest the calcitonin gene-related peptide (CGRP) plays an integral role [4]. Responsible for nociception within the trigeminal ganglion, CGRP represents a major point of interest in the development of migraine prophylactic medication. Thus, numerous studies have emerged within the past decade investigating the effectiveness of monoclonal antibodies (mAbs) as CGRP receptor antagonists in migraine treatment [5].

Older treatment options for migraine prevention have not been very successful in alleviating the personal and economic burden of migraine [6]. A major reason for the lack of success is their limited tolerability and patient adherence [7]. Antiepileptics, beta-blockers, and antidepressants are examples of these medications. Furthermore, these medications have not been very effective in treating migraine headaches and reducing migraine burden [7].

On the other hand, mAbs result in better treatment outcomes and, due to their long half-lives, they can be dosed at long intervals, which can be a preferable option for some patients [8]. Less frequent dosing can minimize the burden on the patient and assures better treatment adherence. Presently, a small collection of monoclonal antibody medications has been approved for the preventative treatment of episodic and chronic migraine by the U.S. Food and Drug Administration (FDA). Such monoclonal antibodies include galcanezumab, eptinezumab, and fremanezumab, which target the CGRP ligand, and erenumab, which targets CGRP receptors, as shown in Table 1 [4].

Recent studies examining the usage of erenumab suggest that it can effectively reduce migraine frequency and improve quality of life [9]. In a similar study assessing the efficacy and tolerability of erenumab among 418 patients, 168 (69.7%) reported that the benefits of erenumab outweighed any potential drawbacks [10]. Episodic migraine patients with at least one previous preventative treatment failure (PPTF) exhibited significantly greater gains in efficacy compared to placebo [11]. Among the aforementioned mAbs, Eptinemzumab stands out as the sole drug capable of intravenous administration, allowing for a rapid onset of action [12]. This unique quality allows for quicker headache pain relief [13] and reduced monthly migraine days compared to placebo [14]. The existing literature on CGRP monoclonal antibodies (CGRP mAbs) suggests high patient tolerability; discontinuation is most commonly attributed to lack of efficacy rather than adverse effects [15]. Despite a lack of major differences in efficacy across clinical trials, a few case studies found that some patients with suboptimal response to one mAb had managed to successfully switch to another one with noted significant improvements [16,17,18,19]. One real-world analysis study demonstrated that one-third of erenumab non-responders achieved >30% response after switching to another CGRP mAb [20]. In the aforementioned analysis, the focus was on switching from erenumab to either galcanezumab or fremanezumab. This was based on the fact that erenumab was approved first in Europe.

These findings warrant further investigation into the efficacy and safety of switching between CGRP mAb classes. Therefore, this study investigated the treatment effectiveness, tolerability, and adherence of migraine patients in the United Arab Emirates (UAE) following a switch from a PPTF to another mAb. The primary objective was to retrospectively assess the reduction in the frequency of monthly migraines headache days and determine the efficacy of the second preventative therapy following the switch from a previously failed CGRP mAb. Additionally, the study sought to evaluate the tolerability of this therapy and report any potential adverse reactions. This study will serve as an integral step toward advancing our understanding of migraine treatment. It shall also represent the first study of its kind by focusing on an underrepresented population in clinical research. 

## 2. Patients and Methods

This was a retrospective, real-world exploratory study. Data used were gathered from one site, namely the American Center for Psychiatry and Neurology (ACPN), Abu Dhabi, UAE. A total of 391 patients with episodic migraine (EM) or chronic migraine (CM) who had received at least one dose of GCGRP/R mAb (erenumab) or CGRP/L mAb (eptinezumab or galcanezumab) were reviewed for eligibility to be included in the study. Fremanezumab is currently not available in the UAE and was therefore not included in the analysis.

Data were gathered from patients’ clinical records, which contain all the required demographic information as well as information relating to diagnosis, medication history, Monthly Migraine Day (MMD) at baseline, visual pain scores, and follow-up visits. Additionally, patient satisfaction with medication was documented in their clinical records. Patients were asked to keep a record of attacks and symptoms in their headache diaries. Efficacy of the prescribed treatment was evaluated by measuring the change in MMDs between visits. Safety was also assessed; this was achieved by monitoring adverse events.

Follow-up visits were scheduled monthly or as deemed necessary, which is a standard protocol at our site for all patients initiating treatment with mAbs. Patients were assessed on their baseline frequency of MMDs and subsequent thorough discussions with treating physician on which mAb would be most effective, addressing each patient’s specific needs. If the current medication did not result in any meaningful reduction in MMDs, an option to switch to another mAb was offered to the patient. The intensity of the headache was also recorded using a visual scale pain, a measurement which was conducted by the treating physician.

The retrospective analysis mainly focused on two main periods. The first period included data from patients treated with a specific CGRP mAb, while the second period involved data from patients who switched to another anti-CGRP mAb. Switching was mainly due to lack of efficacy; it was ensured that patients included in the analysis completed at least 3 months of treatment before switching. Evaluating efficacy after a minimum of 3 consecutive months of treatment was adopted based on the EHF and our local guidelines [21,22]. During each phase, patients’ MMDs were assessed at 3 stages prior to and following their medication switch: at least one month before the first injection (baseline), at a 3-month follow-up, and at a 6-month follow-up.

This study was conducted in accordance with the Helsinki Declaration of 1964 and is consistent with Good Clinical Practice (GCP) guidelines. All ethical guidelines, health authority regulations, and data privacy laws were adhered to. Prior to the start of the study, all relevant approvals were obtained from the ACPN’s Institutional Review Board (IRB), and a waiver of informed consent from the corresponding ethics committee was obtained. To ensure transparency and accuracy, all authors were provided access to the study data. 

**Figure 1:**
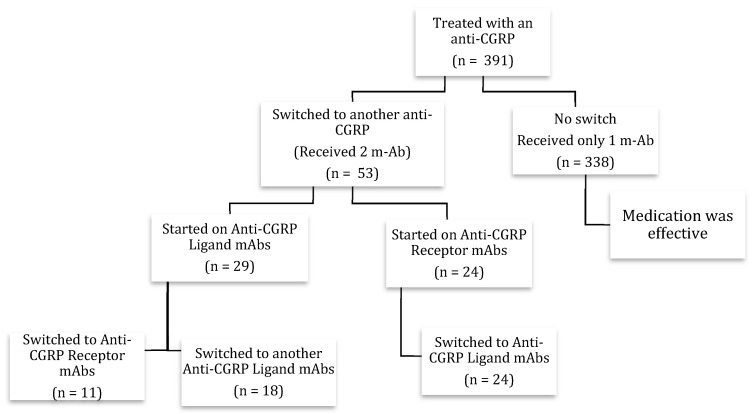
Flowchart of patients.

Records from the ACPN’s nursing department were gathered, and all patients who had been administered one mAb were screened and identified (Figure 1). Data included patients from January 2018 up to September 2022 who were adults (≥18 years) and who had a diagnosis of either EM or CM, as per the International Classification of Headache Disorders (ICHD-3) criteria [23]. Data were shared independent of treatment effects or cause for the CGRP mAb switch. 

Patients were included in the analysis if they (i) switched between two of the previously mentioned CGRP mAbs (switchers), (ii) received at least three doses of the first CGRP mAb, and maintained treatment for a minimum of 6 months after switching. Those who demonstrated a meaningful response (which is defined as more than a 50% reduction in MMDs for EM and more than 30% for CM) and were satisfied concerning their treatment remained on their current preventative treatment and were thus not included in the effectiveness analysis. Patients were categorized according to their switching profiles. The three profiles represented were as follows: CGRP/R mAb to GCRP/L mAb, CGRP/L to CGRP/R, or CGRP/L to another CGRP/L mAb (Figure 1).

The number of MMDs was extracted from the headache diaries, as documented on the patients’ electronic medical records (EMRs). Due to the non-standardized headache diaries and the varying details of documentation of headache characteristics and accompanying symptoms during each headache attack, reliable differentiation between headache and migraine days was not possible. The primary endpoint was the absolute change from baseline in MMD response rate (>25%, >50%, >75%, and 100% reduction in MMDs) for each category of the switchers. As per the methodology outlined by Kaltseis et al. in 2023, patients who demonstrated a positive response to treatment were classified as responders [24]. This was determined by a minimum reduction of 50% in MMDs for EM or a minimum reduction of 30% in MMDs for CM after receiving treatment for a minimum of 3 months. Patient characteristics included age, gender, migraine diagnoses, migraine years, and the type of CGRP mAb from the EMR.

### Statistical Analysis

Since this analysis was conducted retrospectively, the sample size was not based on any statistical consideration. The sample size was achieved depending on the number of cases fulfilling the inclusion criteria treated at ACPN. Continuous variables were summarized using mean ± standard deviation [SD] or median interquartile range [IQR], while categorical data were presented as numbers and percentages. The normality assumption was evaluated using the Shapiro–Wilk test. Given that the data did not follow a normal distribution, the Wilcoxon signed rank test was used to analyze the changes in quantitative variables before and after changes. A significance level of *p* < 0.05 was considered statistically significant for all variables. The statistical software SPSS version 26.0 (IBM Corp. SPSS Statistics, Armonk, NY, USA) was utilized for all data analyses.

## 3. Results

### 3.1. Demographics and Baseline Characteristics

The participant pool was composed of 53 individuals, all of whom had undergone a switch from one CGRP mAb to another; the descriptive statistics for this cohort are visualized in Table 2. Among the 53 participants, 42 (79.2%) of whom were female, 20 (37.7%) were diagnosed with CM, while the remaining 33 (62.3%) were diagnosed with EM. The mean age (SD) of participants in years was 39.2 (11.0). Furthermore, patients were categorized according to their switching profile and had the following distribution: CGRP/L to R mAb (n = 11; 20.7%), CGRP/R to L mAb (n = 24; 45.3%), and CGRP/L to L mAb (n = 18; 34.0%). All 53 patients were diagnosed with migraine headaches without aura.

**Figure 2:**
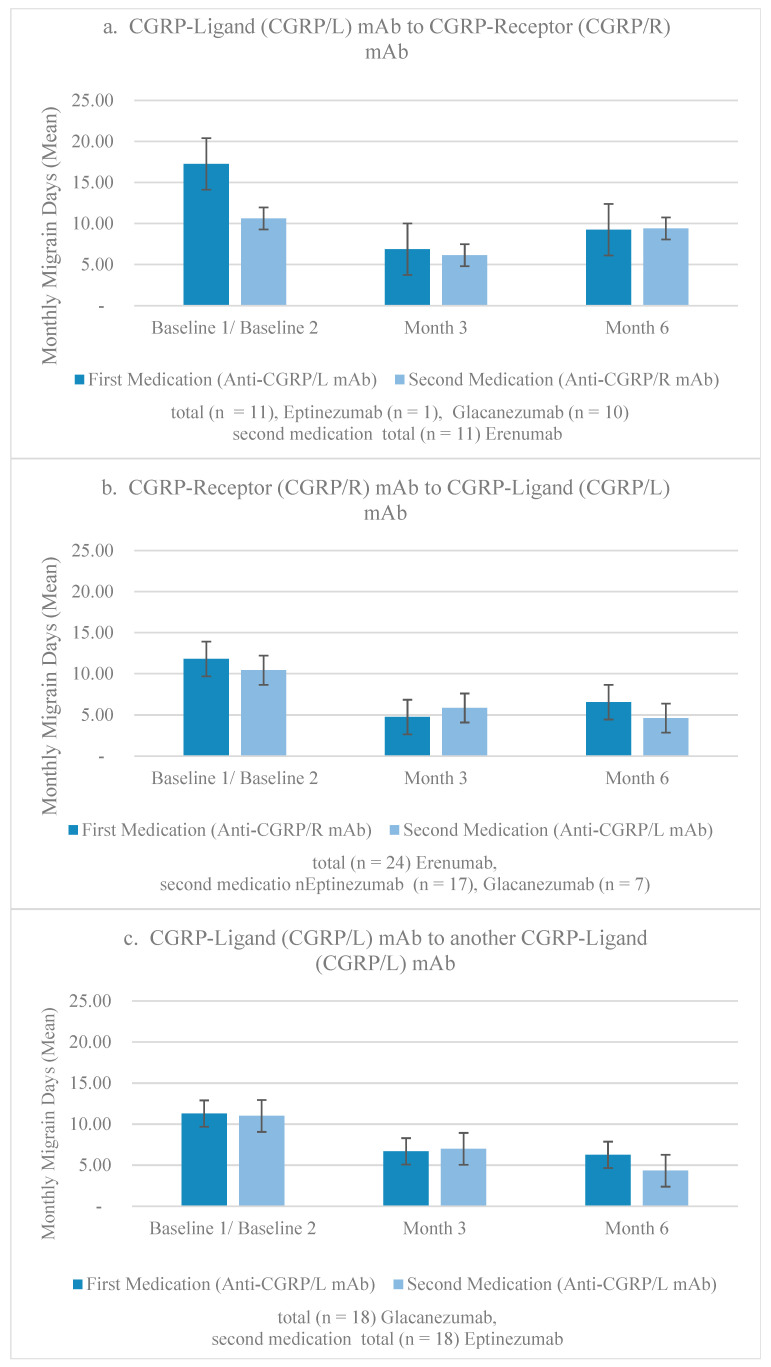
Mean MMDs of switchers. (**a**) MMDs of 11 patients who switched from CGRP/L to CGRP/R mAb. (**b**) MMDs of 24 patients who switched from CGRP/R to CGRP/L. (**c**) MMDs of 18 patients who switched from a CGRP/L to another CGRP/L mAb.

Throughout the study, the frequency of participants’ monthly migraine days (MMD) was assessed at three stages prior to and following their medication switch: at baseline, at a 3-month follow-up, and at a 6-month follow-up (this can be visualized in Figure 2). As exhibited in Table 3, the greatest mean (SD) MMD values were recorded at the baseline assessments; however, it is worth mentioning that the post-switch baseline mean of 10.21 (5.42) was lower than that of the pre-switch baseline mean of 12.92 (8.23). Following the pre-switch baseline assessment, the mean was reduced to 5.53 (5.73) by month 3; however, it rose slightly to 5.92 (6.69) by month 6. Following the post-switch baseline assessment, mean MMDs dropped to 5.74 (4.32) by month 3 and further decreased to 5.42 (4.98) by month 6. Patients began treatment on CGRP mAbs, as suggested by our local UAE guidelines, based on at least 4 MMDs, or fewer if the attacks were severe or disabling based on the Migraine Disability Assessment (MIDAS) or Headache Impact Test-6 (HIT-6) scores [22]. There was a minimum of a 3-month washout period prior to switching. 

### 3.2. First CGRP Monoclonal Antibody

**Figure 3:**
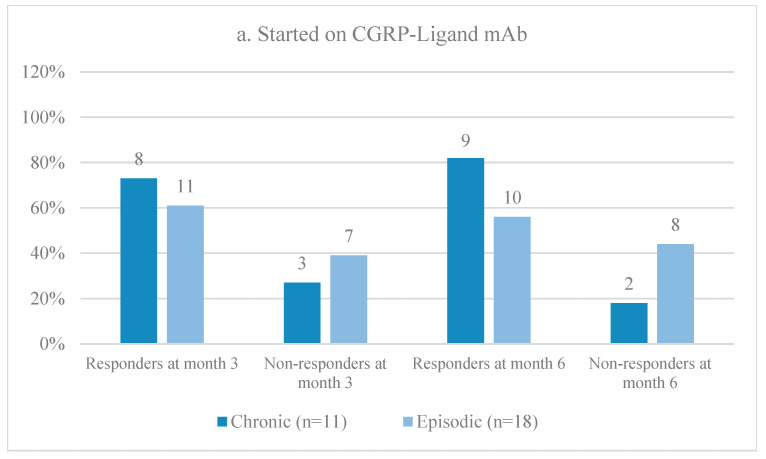

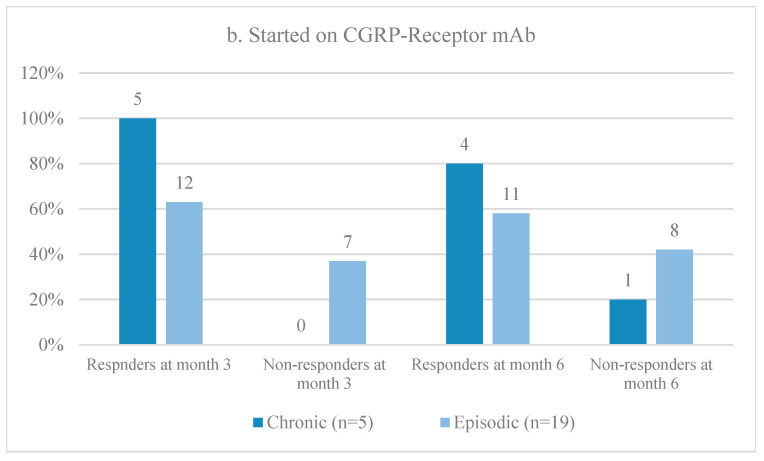
Responder and non-responder rates at month 3 and month 6 for patients with EM or CM on (**a**) CGRP/L mAb and (**b**) CGRP/R mAb during the first observational period.

A total of 29 patients were initiated on CGRP/L mAbs, while 24 patients were started on CGRP/R mAbs. Upon conducting a follow-up at month 3, it was observed that patients with CM exhibited a higher response rate compared to patients with EM. Specifically, 72% of CM patients responded positively to treatment with CGRP/L mAb, whereas 100% of CM patients responded to CGRP/R mAb. On the other hand, 61% and 63% of EM patients responded to CGRP/L mAb and CGRP/R mAb, respectively. However, it is worth noting that the CM group displayed higher rates of non-responsiveness when treated with CGRP/L mAb (27%) as opposed to CGRP/R mAb (0%). Upon analyzing the 6-month follow-up data, no significant improvement in response rates was observed. Notably, 8 EM patients remained non-responders to both classes of CGRP mAb. Further details regarding response rates during the initial observational period on the first medication are displayed in Figure 3.

### 3.3. Second CGRP Monoclonal Antibody

**Figure 4:**
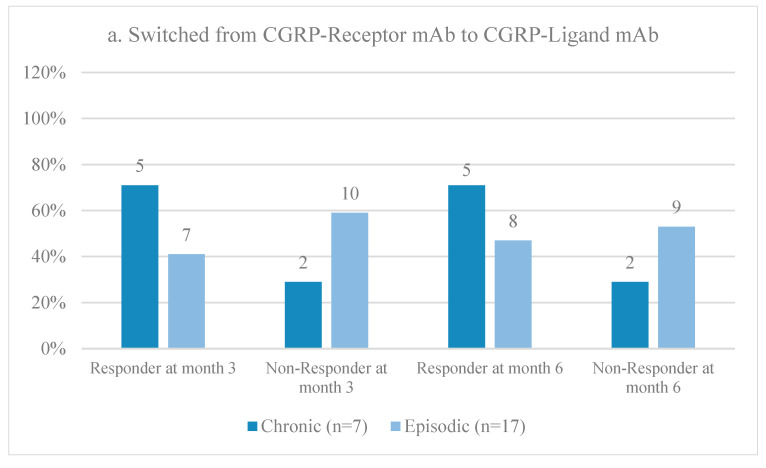

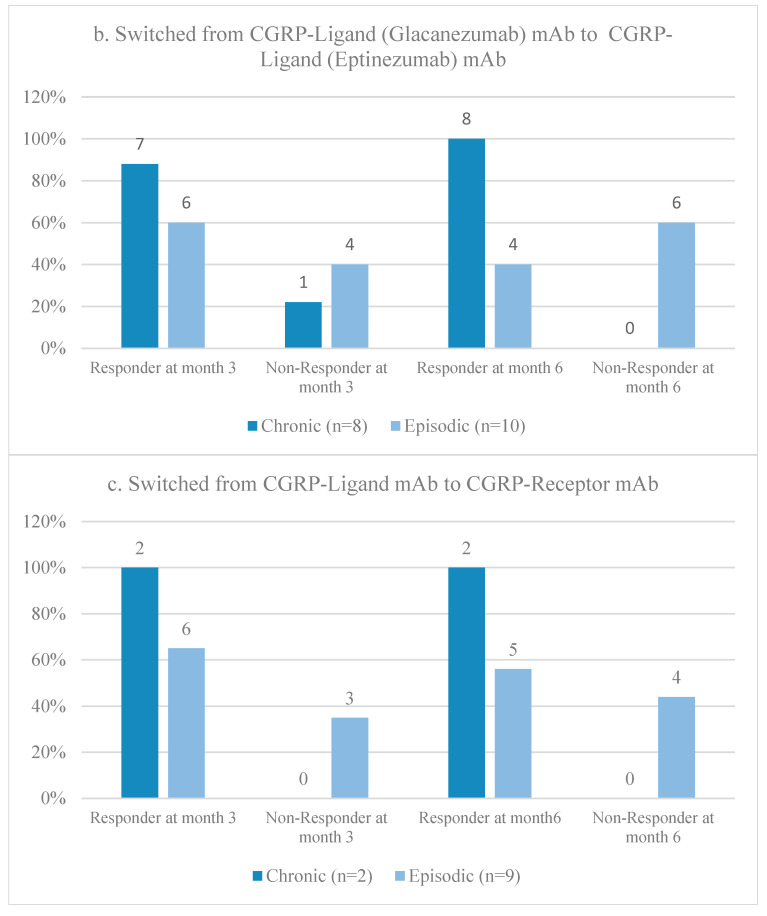
Responder and non-responder rates at month 3 and month 6 for patients with EM or CM (**a**) after switching from CGRP/R mAb to CGRP/L mAb, (**b**) from CGRP/L mAb to CGRP/L mAb, (**c**) or from CGRP/L mAb to CGRP/R mAb.

During the second observational period, the 24 patients who received CGRP/R mAb as their first CGRP mAb switched to anti-CGRP/L mAbs (7 galcanezumab and 17 eptinezumab), whereas out of the 29 patients who started on CGRP/L mAb (28 galcanezumab and 1 eptinezumab), 11 patients were switched to CGRP/R mAbs (erenumab) and 18 patients to another class of CGRP/L mAb (galcanezumab). All the patients in the latter group switched from galcanezumab to eptinezumab, while the one patient who initially received eptinezumab was switched to erenumab. Surprisingly, response rates during the second observational period at the month 3 follow-up dropped in the CM and EM patients who were switched from CGRP/R mAbs to CGRP/L mAbs, specifically from 100% and 63% to 71% and 41%, respectively (Figure 3b and Figure 4a). On the other hand, CM patients who started on CGRP/L mAb had a higher response rate when they were switched to another anti-CGRP/L mAb (100%) rather than to an anti-CGRP/R mAb (87.5%) at month 3 (Figure 4b,c). Overall, CM had a better response rate than EM during the second observational period (Figure 4). 

### 3.4. First versus Second CGRP Monoclonal Antibody

Table 4 and Table 5 present the median differences in MMDs at 6 months compared to the baseline. These tables specifically focus on the first and second CGRP mAbs administered to different patient groups. Interestingly, the overall reduction in MMDs for all patients is precisely identical for both the first and second mAbs.

As shown in Table 6, patients were assessed on their reduction in MMDs from the 6-month follow-up before the switch to their 6-month follow-up after the switch as an additional evaluation of treatment efficacy following a medication switch. In line with the results of the Wilcoxon tests, among ligand–receptor switchers, only one (11.1%) participant experienced a greater than 50% reduction in MMDs, while the vast majority (66.7%) experienced less than a 25% reduction. Although a greater proportion of receptor–ligand switchers (19.1%) experienced a greater than 50% reduction in MMDs, an overwhelming majority (71.4%) experienced less than a 25% reduction. Interestingly, the ligand–ligand cohort exhibited the greatest proportion (33.4%) of participants experiencing a greater than 50% reduction, as well as the lowest proportion (44.4%) of those experiencing less than a 25% reduction. Table 7 and Table 8 represent migraine pain severity and the overall improvement status after treatment respectively. Switching the route of administration from subcutaneous to intravenous could possibly influence treatment outcomes. This shift in administration route may impact pharmacokinetics and alter drug absorption, which may contribute to a better treatment response. It could also influence the patient’s perception of treatment efficacy as a result of enhancing the placebo effect.

### 3.5. Exposure and Safety

In this particular study, a total of 53 patients were included. Out of these patients, it was observed that four individuals had an adverse event (AE), three of which took place prior to the switch in treatment. However, it is important to mention that despite these AEs, these patients did not opt to discontinue the treatment, indicating that the AEs were of a minor nature. AEs included constipation, slight pain on injection site, and increased itchiness. The remaining patient reported an AE after the switch. This particular AE involved peri labial numbness and swelling, which occurred during the infusion. The patient was closely monitored and subsequently discharged safely. Treatment was well tolerated among the remaining 49 patients.

## 4. Discussion

Despite the recent growth in CGRP mAb use as a unique migraine treatment strategy, relatively little is known regarding how switching between CGRP mAb classes can impact the efficacy and tolerability of treatment. Therefore, this study represents another step in furthering our understanding of how CGRP mAb treatments can be safely and effectively applied in a clinical context. Moreover, to our knowledge, this is the first real-world study of its kind to be conducted in the United Arab Emirates and GCC region, thus including an underrepresented population in pivotal trials. Determined prior to data analysis, our primary endpoint was to determine the impact, if any, of switching between CGRP mAb medications on the effectiveness of MMD reduction. Furthermore, our secondary endpoint was to assess the safety and tolerability associated with that switching. 

The 53 patients included in data analysis were classified according to their switching profile, including switching from receptor-targeted to ligand-targeted treatment (RL), from ligand-targeted to ligand-targeted (LL) treatment, and from ligand-targeted to receptor-targeted (LR) treatment. Using a Wilcoxon signed rank test, groups were compared at baseline (BL), month 3 (M3), and month 6 (M6) to assess if changes in MMDs were attributable to treatment. Although RL and LL patients experienced significant reductions in MMDs between BL and M6 after switching, this was not the case for LR patients. This incongruence between groups warrants further investigation with larger cohorts into the pathophysiology of CGRP mAb treatments in order to explain why switching in one direction produces starkly different outcomes than the other.

Indeed, prior to conducting the study, we expected switching between different classes of CGRP mAbs could yield improved results, especially for those switching from L to R or vice versa. However, to our surprise, our results showed that switching from ligand to ligand produced better outcomes. This unexpected finding underscores the importance of studying the mechanism of CGRP mAb treatments in greater detail. Conducting future studies with larger cohorts would provide a better insight into the pathophysiology of these treatments and may help further explain why switching from ligand to ligand produces a better outcome.

As part of our analysis, the Wilcoxon signed rank test included comparisons between M6 MMD data prior to and post switching. Across all switching profiles, there was no significant difference between M6 mean MMDs. There are two perspectives from which to interpret this outcome. On one hand, it is surprising that, even after switching medications, patients did not experience further significant improvements in their MMD reduction when compared to their previous medication. This could lead one to assume that if a patient is discontinuing a CGRP mAb treatment due to lack of effectiveness, it may not be significantly advantageous for them to consider switching to another CGRP mAb medication. However, it is simultaneously encouraging that the failure of a previous CGRP mAb medication due to side effects or poor tolerability may not necessarily suggest that future trial of another mAb would result in the same outcome. 

Despite a lack of statistically significant differences between M6 values before and after switching, we found it valuable to quantify the percentage of MMD reduction according to switching profile. Notably, 33.4% of LL patients experienced at least a 50% reduction in MMDs 6 months post switch when compared to their 6-month data from their initial medication. Among LR and RL patients, less than 20% of each group experienced a similar degree of MMD reduction. These findings offer modest support to the notion that considering drug mechanism of action may lend itself to improved outcomes post switching. Unfortunately, as erenumab is the only drug included that targets CGRP receptors, there is no means of assessing a receptor–receptor switch for a similar phenomenon. 

On the topic of safety, three of four documented AEs took place prior to switching, all of which were considered mild and did not impact patients’ continuation of treatment. It is worth noting that despite experiencing AEs on their previous CGRP mAb treatment, the aforementioned three patients switching to another mAb did not result in worsening or new AEs. This is a promising development pointing to switching as a safe, tolerable, and, probably, effective process for those experiencing side effects with their first CGRP mAb prescription. 

We have identified two retrospective studies examining lateral switching between CGRP mAb therapies [20,21] to serve as points of comparison with our findings relative to those in the existing literature. The study by Overeem et al. [20] conducted a real-world, multicenter analysis of 78 patients with PPTF on erenumab (receptor-targeted therapy) who switched to ligand-targeted therapies. Unlike the 50% meaningful response rate that was used in our study, their analysis yielded a >30% reduction in MMDs by month 3 after switching in 32% of patients and a >50% reduction in 12% of patients. 

The study by Kaltseis et al. [20,21] conducted a larger retrospective assessment of 171 patients who received either one, two, or three different anti-CGRP mAbs. In contrast to the study by Overeem et al., non-response was set as a <50% reduction in MMDs in EM patients and a <30% reduction in CM patients. In total, 5.3% of participants discontinued treatment due to negative side effects. Compared to our study, that study was heavily focused on the quantity of PPTFs. Our study, however, carefully analyzed the impact of switching directionality on outcomes.

In addition, and in contrast to the study by Overeem et al. [20] where patients who switched from a CGRP/R mAb to any CGRP/L mAb experienced improved MMDs, our study did not observe similar outcomes, which could be due to the different characteristics of our cohorts compared to theirs.

When it comes to responding to treatment, it is important to consider that individuals respond differently. There are those who respond promptly, and there may also be individuals who respond later to treatment (late responders and ultra-late responders). A study by Barbanti et al. defined responders as patients with a ≥50% reduction in MMDs at weeks 9–12 from baseline and late responders as those who had a reduction in MMDs between weeks 13 and 16. Ultra-late responders displayed a reduction in MMD between weeks 21 and 24 [25]. This underscores the importance of carefully recognizing this possibility at the time we consider switching patients to a new class of mAbs if they fail their initial mAb therapy. 

Our study had several limitations that must be acknowledged. Being retrospective in nature means that controlling certain variables that may have affected the results was not possible. Additionally, the sample size included was small, which limits the generalizability of our findings. As a result, it is worth noting that the data analyzed involved one patient who was initially on eptinezumab, and further studies may consequently be required to confirm our findings on ligand–ligand lateral switching interactions and to verify the generalizability of the data. Furthermore, we realize that in our study we allowed patients to consider switching to a new class of mAbs after a minimum of 3 months of treatment. We adopted this approach based on the EHF and our local UAE guidelines and following a thorough and shared decision process involving the patient and the treating physician. However, we acknowledge that there is a subset of patients who might be late responders and that a longer duration of initial treatment might have resulted in better response [25]. Furthermore, the effectiveness outcome was solely based on MMD reduction. Thus, it may not fully capture the impact of the intervention on other patient related outcomes. Due to the relatively short observation period of six months for both the first and second medication, it is not possible to rule out the potential for further improvement that could have been attained if patients had prolonged their use of the initial medication before switching to an alternative one. Furthermore, we lack information regarding the potential occurrence of relapse after six months on the second medication following the switch. Lastly, our cohorts included 37 patients who had not previously tried other preventive therapies, representing, probably, less refractory patients than those previously studied in the literature. Out of the 53 patients, 16 individuals had previously attempted preventative treatment for migraine. One patient had tried two preventative therapies before switching to CGRPs mAbs, while the remaining 15 only had one preventative treatment failure prior to switching. Previous preventative treatments included propranolol, amitriptyline, flunarizine, topiramate, and onabotulinumtoxinA. It is important to acknowledge the prescriptions within this region in comparison to other regions across the globe. It is worth noting that currently there exists no prerequisite in this region for individuals to have undergone a specific number of unsuccessful preventative treatments prior to being administered a CGRP mAb.

## 5. Conclusions

This retrospective, real-world exploratory study examining the effectiveness and safety of switching between CGRP mAb treatments serves as an essential step into furthering our understanding of an under-researched topic. The findings of this study suggest that switching from a previous treatment does not significantly impact the new prescription’s effectiveness nor safety.

Among 53 patients enrolled, none experienced significant changes in their MMDs when comparing their mean data for 6 months on the previous medication against 6 months on the new medication. Nevertheless, the data suggest that those switching from one ligand-targeted treatment to another ligand-targeted treatment were more likely to experience additional or compounded reductions in MMDs on top of improvements gained from their initial prescription. 

Overall, the findings present in this study point to CGRP switching as a potentially safe and clinically viable practice that may have applications for those experiencing side effects on their current CGRP mAb. Further research is warranted to better understand the long-term implications of switching beyond a 6-month period, as well as if those switching to a CGRP mAb of the same mechanism are truly likely to experience greater improvements than their counterparts.

## Figures and Tables

**Table 1:** CGRP targeting drugs.

Drug	Mechanism	Indication	Dosing	FDA Approved	Availability in the UAE
Erenumab	Blocks CGRP receptor	Prophylactic	Monthly, subcutaneous	2018	Yes
Eptinezumab	Binds to CGRP ligand	Prophylactic	Quarterly, intravenous	2020	Yes
Galcanezumab	Binds to CGRP ligand	Prophylactic	Monthly, subcutaneous	2018	Yes
Fremanezumab	Binds to CGRP ligand	Prophylactic	Monthly or quarterly, subcutaneous, but intravenous load for cluster headache	2018	No

**Table 2:** Patient demographics and clinical features at baseline. CGRP/L: CGRP ligand; CGRP/R: CGRP receptor.

	Total Cohort
	(n = 53)
Demographics	
Age (years), mean +/− SD.	39.2 +/− 11.0
Sex female, n (%)	42 (79.2%)
Migraine features	
Chronic migraine, n (%)	20 (37.7%)
Pain intensity, mild, n (%)	0 (0.0%)
Pain intensity, moderate, n (%)	48 (90.6%)
Pain intensity, severe, n (%)	5 (9.4%)
Patients with daily headaches, n (%)	10 (18.9%)
Age at migraine diagnosis, mean +/− SD	27.56 +/− 10.62
Migraine duration (years), mean +/− SD	11.6 +/− 11.3
Other features	
Positive family history of migraine, n (%)	16 (30%)
Received prior preventive treatment, n (%)	16 (30%)
Psychiatric comorbidity, n (%)	24 (45.0%)
First monoclonal antibody	
CGRP/L mAb, n (%)	29 (55.0%)
Galcanezumab, n (%)	28 (53)
Eptinezumab, n (%)	1 (2.0%)
CGRP/R mAb (erenumab), n (%)	24 (45.0%)
Second monoclonal antibody	
CGRP/L mAb, n	42
Galcanezumab, n	7
Eptinezumab, n	35
CGRP/R mAb (erenumab), n	11
Switching profile	
CGRP/L to CGRP/R, n	11
CGRP/L to another CGRP/L, n	18
CGRP/R to CGRP/L, n	24

**Table 3:** MMD mean data before and after switching. Total number of patients (n = 53).

Drug	Mean	Minimum	Maximum	Standard Deviation
Pre-switch BL MMD	12.92	1.0	28.0	8.23
Pre-switch M3 MMD	5.53	0.0	28.0	5.73
Pre-switch M6 MMD	5.92	0.0	28.0	6.69
Post-switch BL MMD	10.21	2.0	28.0	5.42
Post-switch M3 MMD	5.74	0.0	15.0	4.32
Post-switch M6 MMD	5.42	0.0	28.0	4.98

**Table 4:** Changes in MMDs during the first CGRP mAb from the first baseline.

	Median Difference (IQR) 6 Month	N
All patients *p*-value	−5.1 (5.5) <0.001	53
Anti-CGRP/L before switching to anti-CGRP/R *p*-value	−2.2 (7.0) 0.025	11
Anti-CGRP/R before switching to anti-CGRP/L *p*-value	−3.15 (4.5) 0.002	24
Anti-CGRP/L before switching to another anti-CGRP/L *p*-value	−3.21 (5.25) 0.001	18

IQR, interquartile range.

**Table 5:** Changes in MMDs during the second CGRP mAb from the second baseline.

	Median Difference (IQR) 6 Month	N
All patients *p*-value	−5.0 (5.0) <0.001	53
Anti-CGRP/R after anti-CGRP/L *p*-value	−1.60 (9.0) 0.109	11
Anti-CGRP/L after CGRP/R *p*-value	−3.73 (3.75) <0.001	24
Anti-CGRP/L after another anti-CGRP/L *p*-value	−3.47 (7.25) 0.001	18

**Table 6:** M6–M6 percent reductions.

Switching Profile	Q1 Frequency (%)	Q2 Frequency (%)	Q3 Frequency (%)	Q4 Frequency (%)
Ligand–Receptor	6.0 (66.7)	2.0 (22.2)	0.0 (0.0)	1.0 (11.1)
Receptor–Ligand	15.0 (71.4)	2.0 (9.5)	1.0 (4.8)	3.0 (14.3)
Ligand–Ligand	8.0 (44.4)	4.0 (22.2)	3.0 (16.7)	3.0 (16.7)

Q1: <25% reduction in MMDs; Q2: 25% < x < 50% reduction in MMDs; Q3: 50% < x < 75% reduction in MMDs; Q4: >75% reduction in MMDs.

**Table 7:** Migraine pain severity.

Pain Severity	Initial n (%)	Final (%)
Severe	5 (9.4%)	3 (5.7%)
Moderate	48 (90.6%)	39 (73.6%)
Mild	0 (0.0%)	11 (20.8%)

**Table 8:** Overall improvement status after treatment.

Status	Number of Patients	Percentage
Improved	37	69.8%
No improvement	15	28.3%
Worsened	1	1.9%

## Data Availability

All data generated or analyzed during this study are included in this published article. No data repository is available.
